# The Baltimore Dentists and the Census

**Published:** 1890-11

**Authors:** 


*Editor of the American Journal of Dental Science :*

### Editorial, Etc.

THE BALTIMORE DENTISTS AND THE CENSUS.—Many of the dental practitioners of Baltimore City have protested against the questions propounded by the special agents who are collecting manufacturing statistics, and several of them have visited Washington to endeavor to convince Superintendent Porter that dentistry is a branch of medicine, and not a manufacturing interest; but, apparently, without success. Dr. Richard Grady, in an article prepared for a daily paper, represented this case as follows: "The prevailing idea that the gold filling and the artificial denture are as legitimate objects of barter and contract as any other tangible object of manufacture has led the censue office at Washington to interpret the term "mechanical dentists to embrace all branches of the profession except the extraction of teeth." Mercantile relations of cost and price are capable of definite adjustment when applied to commodities of known values enhanced by labor at given rates, but professional service is amenable to no such standard. Dentistry is the science and art of medicine applied to the dental organs, and the fee is a valuation of thought and skill. The anatomy, physiology and pathology of dentistry differ in no respect from that taught in medical schools. Dental therapeutics, however, includes a class of operations not taught in medical schools and not practiced in the offices of physicians and surgeons. Hence by universal consent this branch of therapeutics, under the name of dental surgery, is assigned to a special class of practitioners.

"The distinguishing feature of dental therapeutics is the art of replacement—replacement of dental *structure*, replacemet of dental *organs*, which may be classified as dentistry of the chair and dentistry of the laboratory. As medicine and surgery are combined in the practice of the majority of medical men, so the two classes of prosthetic mechanism are usually practiced together, and such practice is in a larger number of cases unavoidable. The element of mechanism and the necessity for the exercise of handcraft enter more or less into all special sciences. Astronomy, chemistry, pharmacy, microscopic analysis and modern medical diagnosis demand extreme accuracy of manipulation, and all great discoveries in these sciences displayed the ability not only to use, but also to invent and construct apparatus.

"The universal recognition of the great value of this element in every department of physics has given the scientific world a correct idea of the true dignity of highly-educated mechanical skill—skill without which the physician's art is crippled, surgery becomes impotent and dentistry has no existence. No science or art except chemistry has been so eminently progressive in recent years as dentistry, and it is second in importance and extent of its usefulness to no specialty of the great art of healing. It requires the talent that would make a good physician, the talent that would make a good surgeon, the talent that would make a good mechanic, and the talent that would make a good artist."

Acting Superintendent Childs, of the census office, decided practically that the protest of the Baltimore dentists against the questions being propounded by the special agents who are collecting the manufacturing statistics is without sufficient basis to justify a compliance with their requests to be left out of the list of manufacturers to be enumerated. According to Mr. Childs and Mr. Williams, the chief of the manufacturer's division, the reluctance on the part of the dentists to answer questions arises from a misapprehension concerning the use to which the information they are required to give is to be put. In the compilation of the statistics no names are to be used, nor any information that could affect the interests of any individuals.

Dr. Grady thereupon replied as follows: "The interpretation given by Mr. Williams to the term mechanical dentistry, namely, 'That branch of the profession which includes the manufacture of artificial dentures, such as plates, crowns, caps,' modifies that given September 23, 1890, by Chief of Division Boudinot to Chief Special Agent Creager: 'You are informed that the term is considered to embrace all branches of the profession except the extraction of teeth.' The census office is in error in claiming that 'at the census of 1880 * * * opposition was uniformly withdrawn when proper explanations * * * were made to the persons averse to giving the information required.'

"The facts are that the superintendent of the census of 1880, upon the representation made to him by the chief special agent of New York 'of the general reluctance of the profession to give the information contemplated by the schedule,' and upon the presentation to him of a 'protest against the novel and unjustifiable demand made for reports from dental practitioners,' determined to omit from the tabulations 'dentistry in *all* its branches.' If, as the result of this decision, all the schedules in the New York office were 'indorsed in this effect and respectfully returned to their respective makers,'

where did the tenth census get the various statistical items relating to mechanical dentistry? Again, referring to the figures of that census, the number of establishments noted for Baltimore is 14, which would imply that but one in ten of 141 dentists whose names are given in the Baltimore City Directory for 1880 manufactured artificial dentures, crowns, caps, &c., whereas it is well known that such work is done by the majority of the profession."

A meeting of Maryland dentists was held Nov., 1890, at which it was determined to resist such action as that contemplated by the census officials, and the following is the latest phase of the matter:

The determination on the part of the Maryland dentists to refuse the information requested of them by the manufactures division of the census office, on the ground that dentistry is a liberal profession and in no sense a manufacturing business, is another thorn in the side of Superintendent Porter. He had not anticipated further trouble after the explanations made by Chief Clerk Childs regarding the matter several weeks ago. Concerning the dentists' contention that dentistry is a branch of the healing art, and not a manufacturing business, Mr. Porter said: "I know that just as well as they do, and so far as the census office occupation tables are concerned, all dentists will be classified as professional men." The objections which were being urged by dentists against including as manufactures any part of their operations seemed, he said, to arise from a misapprehension or misunderstanding of the real object or intention of the office. It is not the purpose of the office to include in the tables of manufactures all operations of the dental profession, but simply the operations of that branch which include the manufacture of artificial dentures, such as plates, crowns, caps, &c., which are manufactured wholly or in part outside of the mouth and afterwards inserted. "There can be no doubt," he claims, "that such features of the profession are clearly within the limits of the census inquiry relating to productive industries, and as such are, under the law, to be included in the tables of manufactures. To omit them would be doing a serious injustice to industries of places like Philadelphia, Baltimore and other cities where this branch of manufactures is such an important element. Not only so, but all this information was collected for the tenth census without a single complaint being made, and included such items as number of establishments, capital invested, number of hands employed, wages paid, materials used and value of products. I feel sure," added the superintendent, "that the dentists of the country, as soon as they understand the purpose of the office in this matter, will see that it is really to their advantage and to the benefit of their profession to answer the questions on the schedule sent to them, and in doing so they cannot in any way impair the high position they now hold among the professional men of this country."

While the census law provides penalties in cases where information is refused in the manner proposed by the Maryland dentists, it is not Mr. Porter's desire, nor does he now believe it will become necessary, to resort to this portion of the law in order to secure the information required. He is sanguine of settling the difficulty without testing in the courts the validity of the census act.

---

